# A probabilistic approach for economic evaluation of occupational health and safety interventions: a case study of silica exposure reduction interventions in the construction sector

**DOI:** 10.1186/s12889-020-8307-7

**Published:** 2020-02-11

**Authors:** Amirabbas Mofidi, Emile Tompa, Seyed Bagher Mortazavi, Akbar Esfahanipour, Paul A. Demers

**Affiliations:** 1 Institute for Work & Health, 481 University Ave Suite 800, Toronto, ON M5G 2E9 Canada; 2 School of Medical Science, Tarbiat Modares University, PO: 14115-111, Tehran, Iran; 3 Department of Economics, McMaster University, Hamilton, Ontario Canada; 4 Department of Industrial Engineering and Management Systems, Amirkabir University of Technology, Tehran, Iran; 5 Occupational Cancer Research Centre, Toronto, Ontario Canada

**Keywords:** Cost-benefit analysis, Lung cancer, Uncertainty, Probabilistic modeling approach, Net benefit, Bayesian networks

## Abstract

**Background:**

Construction workers are at a high risk of exposure to various types of hazardous substances such as crystalline silica. Though multiple studies indicate the evidence regarding the effectiveness of different silica exposure reduction interventions in the construction sector, the decisions for selecting a specific silica exposure reduction intervention are best informed by an economic evaluation. Economic evaluation of interventions is subjected to uncertainties in practice, mostly due to the lack of precise data on important variables. In this study, we aim to identify the most cost-beneficial silica exposure reduction intervention for the construction sector under uncertain situations.

**Methods:**

We apply a probabilistic modeling approach that covers a large number of variables relevant to the cost of lung cancer, as well as the costs of silica exposure reduction interventions. To estimate the societal lifetime cost of lung cancer, we use an incidence cost approach. To estimate the net benefit of each intervention, we compare the expected cost of lung cancer cases averted, with expected cost of implementation of the intervention in one calendar year. Sensitivity analysis is used to quantify how different variables affect interventions net benefit.

**Results:**

A positive net benefit is expected for all considered interventions. The highest number of lung cancer cases are averted by combined use of wet method, local exhaust ventilation and personal protective equipment, about 107 cases, with expected net benefit of $45.9 million. Results also suggest that the level of exposure is an important determinant for the selection of the most cost-beneficial intervention.

**Conclusions:**

This study provides important insights for decision makers about silica exposure reduction interventions in the construction sector. It also provides an overview of the potential advantages of using probabilistic modeling approach to undertake economic evaluations, particularly when researchers are confronted with a large number of uncertain variables.

## Background

Construction workers are at a high risk of exposure to various types of hazardous substances such as crystalline silica [1, 2]. Crystalline silica is an abundant material that is commonly released in respirable form during different construction activities such as concrete work, abrasive blasting, demolition, excavation, earth moving, tunnel construction, and highway building [3]. Reports indicate that the level of silica exposure for numerous construction workers in Ontario, Canada exceed occupational exposure limit (i.e. 0.05 mg/m^3^) [1]. This is likely the case in other jurisdictions across Canada and internationally. Meanwhile, occupational silica-related diseases such as lung cancer annually impose considerable direct costs to the healthcare system and indirect costs to industry in the form of lost output and reduced productivity, as well as high intangible costs in the form of health-related quality of life losses to afflicted workers and their families [4].

There are several silica exposure reduction interventions applicable to construction projects [5–10]. These interventions work in different ways, e.g., preventing silica dust from getting into the atmosphere; removing dust in the atmosphere; and preventing workers from inhaling the dust if present in the atmosphere. Wet method (WM) refers to the use of water with devices to reduce the release of silica dust. Local exhaust ventilation (LEV) refers to the use of local vacuum systems at the point of operation to reduce the release of free silica dust into the work environment. Personal protective equipment (PPE) refers to the use of National Institute for Occupational Safety and Health approved air-purifying or supplied-air respirators. Enclosed work areas and work hygiene practices are some other common types of intervention options, but are not considered here.

Though several studies provide evidence on the effectiveness of different silica exposure reduction interventions in the construction sector, choosing a specific intervention is best informed by an economic evaluation. Despite the importance of the issue, there are only a few economic evaluations of silica exposure reduction interventions. One of these studies by Lahiri et al. [7] evaluates the costs and effects of different interventions for the prevention of occupationally induced silicosis. They estimate the cost-effectiveness in terms of the dollars spent to obtain an additional healthy year. Another economic evaluation study by the Occupational Safety and Health Administration (OSHA) in the United States [5] addresses issues related to costs, technological feasibility, and the economic impacts of the proposed respirable crystalline silica rule, which attempts to reduce the permissible exposure limits from its current level of 0.1 mg/m^3^ to 0.05 mg/m^3^. To do so, the authors forecast the number of silica-related diseases averted as a result of the proposed rule and compare the value of averted cases with the cost of compliance to the rule in all affected industrial sectors.

Uncertainty about the magnitude of input variables of an intervention, which has often been cited as a limitation in economic evaluation studies, can affect the precision of results [11, 12]. Input data for these studies can be provided as probabilistic or deterministic values. Deterministic values should only be applied when specific values are available from a reliable source, while it is best to use probabilistic values when the reliability of information is questionable [13]. In the case at hand, we have large number of uncertain variables that impact an intervention’s economic evaluation results. For instance the number of silica-exposed workers and the level of exposure to silica are uncertain variables. The level of exposure is influenced by several factors such as the task, workstation characteristics (e.g. being indoor or outdoor), materials being used, phase of the construction project and other unknown variables. In many circumstances, it is not possible to collect more data on the level of exposure because of the quick pace of change on a construction project site, tasks characteristic, and/or safety requirements [11]. The risk of getting a silica-related occupational disease for workers of different age and sex also has a high degree of uncertainty, since latent health conditions such as lung cancer are influenced by multiple factors not easily recognized as attributable to occupational silica exposures [14]. The cost of respiratory disease treatment is also an uncertain variable as it depends on, amongst other things, the stage of the disease and the age and sex of the individual [15, 16]. In terms of the effectiveness of a silica exposure reduction interventions, the maximum is achieved by appropriate and systematic use of an intervention, which is not always the case in practice. For example, some studies suggest that the malfunction of PPE is influenced by several environmental factors such as worker’s awareness, the nature of the hazard, climate, and occupational health and safety inspections [17]. The overall effectiveness of WM and LEV interventions also depends on the workstation characteristics and the number of people working near silica dust sources. Because work arrangements vary within occupations and across facilities of different sizes, there is no definitive data on how many workers are likely to be protected by a given intervention [5–10].

There are several probabilistic modeling approaches for solving problems under different levels of uncertainty and estimation of expected value, such as decision trees [18], Markov models [18], and Bayesian networks (BN) [19]. Decision tree analysis involves drawing on a tree-shaped diagram to assist with statistical probability analysis and identifying a solution to the problem. In decision trees, the probability of each possible event is explicitly identified, along with the consequences of those events. This method is frequently used in health economics, specifically for problems that are more complex in nature [18]. Markov models are being used more often in economic evaluation and are probably the most common type of model used in the economic evaluation of healthcare interventions [18]. The main advantage of a Markov model is the representation of recurring events. Although they are similar to decision trees, they do not allow for interaction among variables [18]. BN (also called belief networks) approach, is a graphical structure that allows one to capture the relationships between variables. To illustrate these relationships, a diagram of nodes and arrows is often used. Nodes represent the system variables and the arrows symbolize the direct dependencies among the variables. BN are used to compute the distribution probabilities in a set of variables according to the observation of some variables and the prior knowledge of the others [19]. Recently, the BN approach has gained popularity in different areas of health economics [13], project cost and risk analysis [20–22], cost-benefit analysis [23], and occupational health and safety decision making [24]. BNs are preferred for several reasons, such as the ability to integrate various types of data (i.e., qualitative and quantitative), to combine available data with expert knowledge, to explicitly consider relationships between variables, to model complex problems with many variables involving a high level of uncertainty and to easily provide graphical representations [19–23]. The modeling languages of BNs have straightforward semantics, namely that of cause and effect. Furthermore, the needed probability calculation of BNs is often undertaken with the assistance of software packages such as Netica, GeNIe, BayesiaLab, Analytica, Hugin, Bayes Net Toolbox, and many others (this is not a comprehensive list, and not meant to promote any specific software). In this study, our objective is to identify the most cost-beneficial silica exposure reduction intervention for the construction sector in Ontario, Canada. To estimate the net benefit of each intervention, we apply a probabilistic modeling approach to compare the expected cost of lung cancer cases averted, with expected cost of implementation of each intervention in one calendar year. We anticipate this study provides important insights for occupational health policy makers and workplace parties in the construction sector. More broadly, this study provides a methodological framework for a more complete treatment of uncertainties in the economic evaluation of occupational health and safety interventions via BN.

## Methods

### Study steps

**Fig. 1:**
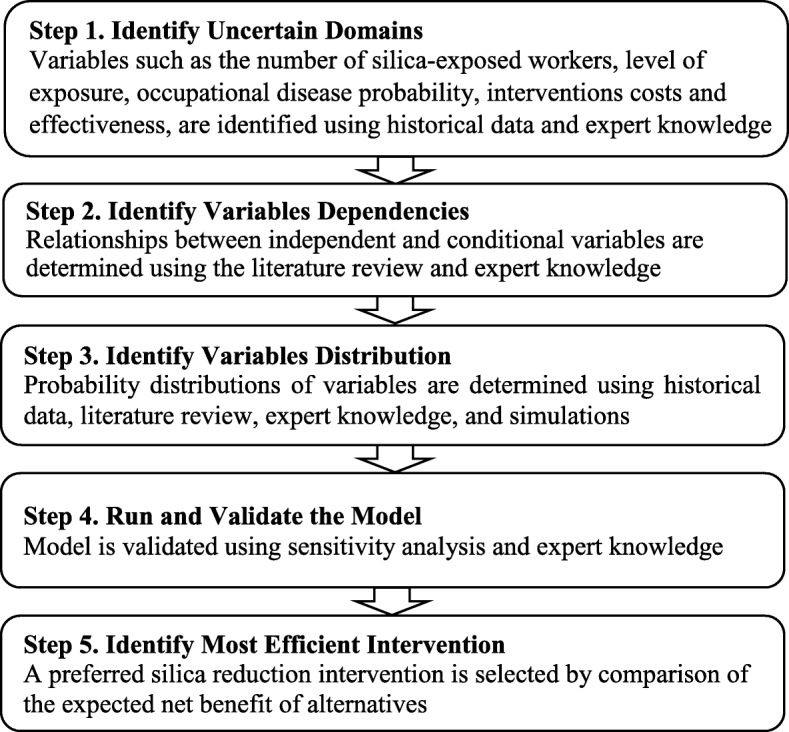
Steps of a Probabilistic Modeling Approach for Economic Evaluation of Silica Exposure Reduction Interventions

Figure 1 identifies the main steps of our probabilistic modeling approach for the economic evaluation of silica exposure reduction interventions. In the first step, we identify all variables that impact the net benefit of the silica reduction interventions, such as the number of silica-exposed workers, level of exposure and intervention effectiveness. Variables not dependent on any other variables (called root nodes in BN vocabulary) have a single probability distribution, whereas variables dependent on one or more other variables (called child nodes) have a conditional probability table (dependency of a child variable to its parent’s variables) [19]. In the second step, we identify dependencies between variables via a literature review and expert knowledge. Six researchers with the following backgrounds were involved in all stages of the project meeting: an expert in silica reduction interventions, two occupational health specialists, one economist, and two epidemiologists. Brainstorming sessions and interviews with experts were relatively unstructured. In sessions, participants were all given an opportunity to contribute to the discussion until consensus was reached. Expert feedback also helps us to identify variables and interactions that were overlooked when first developing the model. In the third step, we identify the probability distributions of variables, drawing on several scientific literatures in epidemiology [25, 26], occupational cancer economic burden studies [4, 16], and silica exposure reduction interventions [5–10]. Once the distributions of independent variables are determined, we compute the probability distributions of conditional variables according to the knowledge of their parents. The main assumptions about the distribution of each variable are explained in the following paragraphs. To develop the structure of BN model and to compute the probability distributions, we use GeNIe modeller version 2.2.4 (BayesFusion, Pittsburgh University decision system laboratory) [27]. Step four involves establishing the structural validity of the model. We validate the model by setting the variables to extreme values and turn to expert judgment to confirm whether the range of results (e.g. expected lung cancer cases, averted costs, and/or interventions costs) appears reasonable. Sensitivity analysis is also undertaken to quantify how different values of independent variables affect the net benefit of interventions. In the fifth and last step, we select a preferred silica reduction intervention by comparing the expected net benefit of alternatives. Benefits are the expected cost of lung cancer cases averted after implementation of different interventions. We use an incidence cost approach and estimate the societal lifetime cost of lung cancer cases. Then we calculate expected net benefit as the difference between the expected benefit from expected cost of each intervention in a calendar year (i.e., 2020). Costs of the intervention are based on the assumption that there is no use of preventive measures at baseline. Economic evaluation is conducted from societal perspective. A discount rate of 3% was used to obtain the present value. All monetary values are converted to 2017 Canadian dollars.

### Input data

To determine the probability distributions of variables, we combine our model assumptions with secondary data drawn from various sources such as the Occupational Cancer Research Centre (OCRC) [25], CAREX Canada [26], Canadian Life Tables [28], the Labour Force Survey (LFS) [29], the Survey of Labour and Income Dynamics (SLID) [30], Canadian System of National Accounts (CSNA) [31], the General Social Survey (GSS) [32], the Canadian Cancer Risk Management Model (CRMM) [33], the Survey of Employment, Payrolls and Hours (SPEH) [34], Canadian Community Health Survey (CCHS) [35], and various scientific published and grey literature sources.

### BN model

**Fig. 2:**
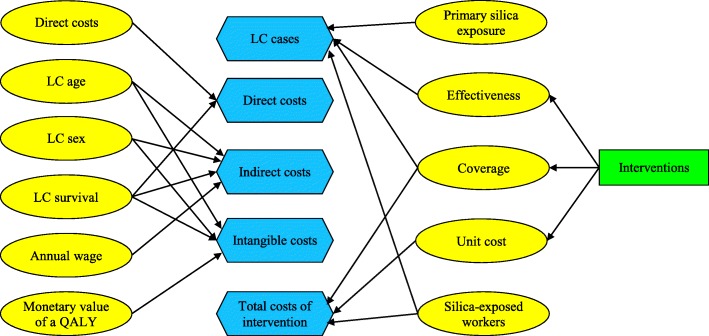
A Simplified Representation of Economic Evaluation Model of Silica Exposure Reduction Interventions, Using Bayesian Network. Note. Ellipses represent random variables, rectangles represent silica exposure reduction intervention options as decision variables, and hexagons represent costs as values or utility nodes, Interventions: wet method (WM), local exhaust ventilation (LEV), personal protective equipment (PPE), and combinates of the following: WM-LEV-PPE, WM-LEV, WM-PPE, LEV-PPE, primary silica exposure: level of exposure to silica dust before an intervention, effectiveness: reduction ability of silica exposure interventions, coverage: percentage of silica-exposed workers that a specific intervention is applicable to, unit cost: cost of implementation of an intervention, direct costs: sum of healthcare, informal care, and out-of-pocket costs of lung cancer cases. Indirect cost: sum of output/productivity losses and home production losses of lung cancer cases, intangible cost: monetary value of health-related quality of the life losses of lung cancer case

A simplified representation of the model is illustrated in Fig. 2 (the full network is provided in Additional file 1: Part A). With this model we estimate the expected cost of lung cancer cases averted given different silica exposure reduction interventions. The silica reduction intervention decisions in the model include one of three interventions of WM, LEV and PPE, as well as the following combinates: WM-LEV-PPE, WM-LEV, WM-PPE, LEV-PPE, gives rise to seven different silica exposure reduction possibilities (represented by rectangles). These are the main silica reduction interventions in OSHA’s hierarchy of controls, after elimination/ substitution [5]. Although, the elimination/ substitution of silica with materials containing less amount of silica is the most effective way to protect workers, we do not consider it in this study, mainly because of the large dependency of the construction sector to silica-containing supplies. In the BN, to demonstrate the uncertainty related to each domain, we use random variables (represented by ellipses). A random variable can assume more than one value due to chance (e.g. sex of lung cancer cases is a variable with two values, i.e., male and female that each value has a probability of occurrence). In our model, the random variables related to the lung cancer case costs are age, sex, survival rate, direct costs of lung cancer cases, annual wage of workers, and monetary value of a quality-adjusted life-year (QALY). The random variables related to the interventions costs are the number of silica-exposed workers in the construction sector, silica exposure level, intervention’s effectiveness, coverage and unit cost. Implementation of each of these interventions bears on the intervention costs, the exposure reduction experienced by workers, and in the long run, on the total number of lung cancer cases and related costs averted. BN uses utility nodes for estimation of the expected costs and benefits of the decision to be made (represented by hexagons). These two types of nodes (i.e., decision nodes and utility nodes) enhance the BN to decision support tool to determine the decision to make, which gains the highest expected utility, considering the given circumstances [19, 23]. Additional file 1: Part B lists variables definition, distribution and data sources.

#### Number of silica-exposed workers and level of exposure

We estimate the number of the silica-exposed workers in the Ontario, Canada construction sector as about 91 thousand, based on estimates from OCRC Canada [25]. (exposed occupations listed in Additional file 1: Part C). We also identify the level of silica exposure among construction workers into three ranges: low (< 0.0125 mg/m^3^), medium (0.0125–0.025 mg/m^3^), and high (> 0.025 mg/m^3^), with probabilities of 0.47, 0.39, and 0.14, respectively, based on occupational exposure data sources from CAREX Canada [26].

#### Intervention’s effectiveness, coverage and cost

Wide ranges of effectiveness have been reported for silica exposure reduction interventions in the literature [5–10]. We identify the lowest reported effectiveness of WM, LEV and PPE at 82% [7], 93% [9], and 90% [7], respectively. However, full effectiveness of interventions is only achieved when they are used under the ideal conditions. For example, WM is fully effective when the system supplies a continuous stream or spray of water at the point of impact, which requires regular filling of the water tank and inspection of hosing and nozzles. Similarly, full effectiveness of PPE is achieved when respirators are used, cleaned and inspected routinely. In construction worksites, the interventions are not always working under ideal conditions. As a conservative assumption, we consider 75% of the reported values, for estimation of interventions effectiveness in the construction projects. For a combined use of each of WM or LEV with PPE (i.e. WM-PPE, LEV-PPE), we consider the additive effects. Level of silica exposure after implementation of intervention is modelled by considering primary silica exposure and the effectiveness of each intervention (Additional file 1: part D).

Some of silica reduction interventions are only applicable to certain occupations in the construction sector. We define intervention coverage, to incorporate this variable into our model. The coverage of WM and LEV are estimated at 60 and 40%, respectively, based on the OSHA [5], which means among all silica-exposed workers in the constructions sector, only these percentages can be protected by each intervention. We assume PPE is applicable to all construction occupations (Additional file 1: part E).

Intervention costs are estimated by using three variables: 1) number of silica-exposed workers that are protected by intervention, 2) intervention unit cost and 3) intervention protection factor, as indicated in expression 1. For estimation of the unit cost of the WM, LEV, and PPE, we use OSHA [5] (Additional file 1: part F). The protection factor represents the number of silica-exposed workers that can be protected by each unit of WM or LEV. Recall, WM and LEV protect a group of workers, so for estimation of the total cost of these interventions, we need to know how many workers are protected by each unit of them. For estimation of the protection factor of both WM and LEV, we drew from Lahiri et al. [7] and estimate their protection factor average at 5 workers, and assume it ranging from 1 to 10 workers with Gaussian distribution. Note that PPE total cost does not depend on the protection factor, as each unit of PPE only protects one silica-exposed worker.

1 $$ \mathrm{Totalcost}{\mathrm{ofintervention}}_{\left(\mathrm{x}\right)}=\frac{\mathrm{intervention}{\mathrm{unitcost}}_{\left(\mathrm{x}\right)}\times {\mathrm{protectedgroup}}_{\left(\mathrm{x}\right)}}{{\mathrm{protectionfactor}}_{\left(\mathrm{x}\right)}} $$

#### Lung cancer cases age, sex, survival

We define the age of occupational lung cancer cases in 13 intervals, ranging from 25 to more than 85 years of age [25]. The highest probability of lung cancer is between 70 and 74 years. This older age of onset is due to the long latency of this disease (Additional file 1: part G). Additionally, men have a higher incidence of occupational lung cancer than women (0.7 versus 0.3) because of their higher level of exposure in different male-dominated occupations in the construction sector [25]. We identified the survival probability of lung cancer cases at 0.09 from CRMM [33].

#### Annual wage of workers

To estimate average labour-market earnings of workers for each age and sex group, we used LFS [29], and SLID [30]. Then we add 14% to account for payroll cost paid by employers, based on employer contribution data from the CSNA [31]. We define labour-force participation following treatment of lung cancer cases at 0.77, similar to Earle et al. [36] It is assumed that once they returned to work, their productivity is the same as the productivity of the general population.

#### Monetary value of a quality-adjusted life-year

Given the wide range of monetary values of a QALY in the health economics literature, we consider a range of value in the form of sensitivity analyses. Our baseline value is $150,000 which is reflective of willingness-to-pay values for a QALY identified in recent studies [37]. For sensitivity analyses we use a range from $100,000, which has been used in Canada in the health technology assessment field, to $200,000 which has been extrapolated from increases in health care spending over time and the health gains that have been associated with those increases [38].

#### Lung cancer cases

The number of lung cancer cases expected to arise from different levels of silica exposure, is estimated by using two variables— the number of the silica-exposed workers that are protected by each intervention, and the probability of lung cancer, as described in expression 2. The number of silica-exposed workers that are protected by each intervention depends on the intervention coverage described above. We estimate the probability of lung cancer for different level of silica exposure ranges from low, medium, and high at 9.1E-4, 1.2E-3, and 1.4E-3, respectively, based on OCRC^.25^ (Additional file 1: part H). After the implementation of each intervention, silica exposure is reduced to a lower level, depending on the effectiveness of the intervention (e.g. by using PPE the level of silica exposure shifts from medium to low) and consequently, we expect a lower probability of lung cancer among the protected group of silica-exposed workers. In the expression, x is the silica exposure reduction from the interventions, which is WM, LEV, PPE, or some combination of them.

Lung cancer cases_(x)_ = number of the workers protected_(x)_ × probability of lung cancer_(x)_(2).

#### Lung cancer direct, indirect and intangible costs

These are three sub-categories of the economic burden of lung cancer cases, which are estimated based on our previous study [16]. We identify the direct cost of lung cancer in three categories: healthcare [33], out-of-pocket costs [39], and informal caregiving costs [40], and assume it follows a Gaussian distribution [41]. We include output/productivity losses and home production losses of lung cancer cases under the indirect cost category, and monetary value of health-related quality of life losses of lung cancer under the intangible cost category. We considered the monetary value of time lost due to poor health or premature death using survival probabilities from the Canadian population [16]. The description of the techniques used to estimate these costs, are presented in Additional file 1: part I.

## Results

### Expected costs and benefits

Table 1 presents the expected lung cancer cases averted and net benefit of the seven silica exposure reduction interventions. The values are calculated separately for each of the seven intervention combinations. The percentage of the silica-exposed workers assumed to be protected by each intervention, and the expected lung cancer cases averted are indicated in the first and the second rows, respectively. In the table, we illustrate the cost of lung cancer cases averted (i.e. the benefit) with a positive sign and the intervention costs with a negative sign. As indicated in Table 1, we find the highest lung cancer cases are averted with a combined use of WM, LEV and PPE, about 107 cases, resulting in a net benefit of $45.9 million. With this intervention, all the silica-exposed workers are simultaneously protected with a combined use of the three methods, which makes the cost of this intervention the highest amongst the seven interventions.

**Table 1:** Expected Costs and Benefits of Silica Exposure Reduction Interventions

Interventions	WM-LEV-PPE	WM-LEV	WM-PPE	WM	LEV-PPE	LEV	PPE
Protected workers^a^	100%	100%	100%	60%	100%	40%	100%
Lung cancer cases averted^b^	107	95	102	55	101	40	96
*Averted costs (benefits)*							
Direct	$9.5 M	$8.4 M	$9.0 M	$4.9 M	$8.9 M	$3.5 M	$8.6 M
Indirect	$41.2 M	$36.6 M	$39.3 M	$21.1 M	$38.8 M	$15.3 M	$37.1 M
Intangible	$133.9 M	$119.1 M	$127.6 M	$68.6 M	$126.0 M	$49.7 M	$120.7 M
Total	$184.5 M	$164.2 M	$175.9 M	$94.5 M	$173.8 M	$68.5 M	$166.4 M
*Intervention costs*							
WM^c^	-$42.0 M	-$42.0 M	-$42.0 M	-$42.0 M	$0	$0	$0
LEV^d^	-$15.5 M	-$15.5 M	$0	$0	-$15.5 M	-$15.5 M	$0
PPE^e^	-$81.1 M	$0	-$81.1 M	$0	-$81.1 M	$0	-$81.1 M
Total	-$138.6 M	-$57.6 M	-$123.1 M	-$42.0 M	-$96.6 M	-$15.5 M	-$81.1 M
Net benefit^f^	$45.9 M	$106.6 M	$52.8 M	$52.5 M	$77.2	$53.0 M	$85.3 M
Benefit to cost ratio^g^	1.3	2.9	1.4	2.2	1.8	4.4	2.1

Note. ^a^Percentage of the silica-exposed workers in construction sector that are protected by each intervention, ^b^expected number of the occupational lung cancer cases averted, ^c^total cost of implementing WM, ^d^total cost of implementing LEV, ^e^total cost of implementing PPE, ^f^difference between cost of lung cancer cases averted and cost of intervention, ^g^calculated by dividing the total benefits by the total costs of an intervention. Due to rounding, columns, and rows may not sum to 100%, All table monetary values are in 2017 Canadian dollars

With simultaneous use of WM and LEV, about 95 lung cancer cases are expected to be averted. With this intervention, all silica-exposed workers are protected via WM or LEV. The net benefit of this intervention is $106.6 million, which is the highest among the seven interventions. The implementation cost of this intervention is much less than the cost of the combined use of all three methods, which makes it a more desirable intervention in the case of budget restrictions.

In the case of WM-PPE or LEV-PPE use, we expect a similar number of lung cancer cases averted, about 102 and 101 cases, respectively. With these interventions all silica-exposed workers are protected by PPE, but only a percentage of them are protected by WM or LEV. For example, in WM-PPE, 60% of the silica-exposed workers are protected by both WM and PPE and the remainder are protected with PPE, while for LEV-PPE only 40% of all silica-exposed workers are protected by both LEV and PPE. The net benefit of WM-PPE is estimated at $52.8 million, which is much lower than LEV-PPE, at about $77.2 million, due to its higher intervention cost.

With PPE use alone, we expect 96 lung cancer cases averted and estimate a net benefit of $85.3 million. The results indicate that lung cancer cases averted with PPE are relatively higher than WM and PPE on their own. However, the total benefit of this intervention is lower than WM and PPE, due to a higher implementation cost.

The lung cancer cases averted with WM and LEV on their own are estimated at 57 and 42 cases, respectively, which is relatively lower in comparison to other intervention options, as they only protect a percentage of the silica-exposed workers (i.e., 60% in WM and 40% in LEV). The net benefit of WM is estimated at $52.8 million, which is slightly lower than LEV, at $53 million, due to its higher intervention cost.

The benefit-cost ratio of all seven interventions are positive. The highest benefit-cost ratio is achieved with LEV (4.4), followed by combined use of WM and LEV (2.9), WM (2.2), PPE (2.1), LEV-PPE (1.8), WM-PPE (1.4), and WM-LEV-PPE (1.3). The general rule of thumb is that if the benefit is higher than the cost, the project is a good investment (i.e., a benefit-cost ratio greater than 1). Although it is important to note this fact, WM and LEV on their own protect only a percentage of silica-exposed workers.

### Sensitivity analysis

Table 2 shows how the number of silica-exposed workers and the level of exposure affect the net benefit of each of silica exposure reduction interventions. For this part, we only evaluated interventions that protect the entire silica-exposed workers, namely WM-LEV-PPE, WM-LEV and PPE. The first column represents outcomes, when all variables are in their default distribution. When none of the interventions are implemented, we expect 110 lung cancer cases, which results in an economic burden of $189 million. We set the level of exposure to low, medium, and high and estimate the net benefit of the interventions, for the lower and upper bound values of silica-exposed workers in the construction sector. With a combined use of the three types of prevention activities, we expect a net benefit of $4 million when we set silica-exposed workers and level of exposure at the lower bound value, while we expect net benefit of $107 million when set at the upper bound value. With WM and LEV combined and PPE on its own, we expect a net benefit of $60 million and $45 million, respectively, when we set the silica-exposed workers and level of exposure at the lower bound. We expect a net benefit of $101 million and $94 million respectively when we set it at the upper bound. Note that WM and LEV combined and PPE on their own both protect 100% of silica-exposed workers.

**Table 2:** Sensitivity Analysis of Interventions for Different Numbers of Silica-Exposed Workers and Different Levels of Exposure

Primary silica exposure	Baseline	Low Exposure (< 0.0125 mg/m^3^)		Medium Exposure (0.0125–0.025 mg/m^3^)		High Exposure (> 0.025 mg/m^3^)	
Silica-exposed workers^a^		Lower bound	Upper bound	Lower bound	Upper bound	Lower bound	Upper bound
	91	46	118	46	118	46	118
*No Intervention*							
Expected LC cases^b^	110	60	111	80	140	95	180
Total LC costs^c^	$189 M	$103 M	$191 M	$138 M	$241 M	$164 M	$310 M
*WM-LEV-PPE*							
LC cases averted^d^	107	60	111	79	138	84	162
Total LC costs averted^e^	$185 M	$103 M	$191 M	$136 M	$238 M	$145 M	$280 M
Total intervention costs^f^	$151 M	$99 M	$173 M	$99 M	$173 M	$99 M	$173 M
Net benefit^g^	$46 M	$4 M	$19 M	$37 M	$65 M	$46 M	$107 M
Net benefit change (%)	–	10%	41%	80%	142%	100%	233%
*WM-LEV*							
LC cases averted^d^	95	60	111	71	124	46	100
Total LC costs averted^e^	$164 M	$103 M	$191 M	$122 M	$214 M	$79 M	$172 M
Total intervention costs^f^	$63 M	$43 M	$71 M	$43 M	$71 M	$43 M	$71 M
Net benefit^g^	$107 M	$60 M	$120 M	$79 M	$143 M	$36 M	$101 M
Net benefit change (%)	–	57%	113%	74%	134%	34%	95%
*PPE*							
LC cases averted^d^	96	59	109	73	129	57	113
Total LC costs averted^e^	$166 M	$101 M	$187 M	$126 M	$222 M	$97 M	$195 M
Total intervention costs^f^	$81 M	$56 M	$101 M	$56 M	$101 M	$56 M	$101 M
Net benefit^g^	$85 M	$45 M	$86 M	$70 M	$120 M	$42 M	$94 M
Net benefit change (%)	–	53%	101%	83%	141%	49%	110%

Note. ^a^number of the silica-exposed workers in the construction sector in thousand, ^b^expected occupational lung cancer cases,^c^total cost of occupational lung cancer cases with no intervention, ^d^expected occupational lung cancer cases averted after implementation of an intervention, ^e^total cost of lung cancer cases averted, ^f^cost of implementing a silica exposure reduction intervention, ^g^difference between cost of lung cancer cases averted and cost of intervention. All table monetary values are in 2017 Canadian dollars

## Discussion

Among the seven silica exposure reduction interventions considered in this study, we estimate the highest number of lung cancer cases are averted with a combined use of WM-LEV-PPE (107 cases). Despite this fact, the highest net benefit is achieved with WM and LEV, about $106.6 million, due to their lower implementation costs. The lowest number of lung cancer cases are averted with WM or LEV (55 and 40 cases), as these interventions protect only a fraction of the silica-exposed workers. With a low or medium level of silica exposure, a combined use of WM and LEV are expected to produce the highest net benefit, while with a high level of exposure, the combined use of WM-LEV-PPE is expected to result in the highest net benefit.

In terms on future uses of BN in the area of Occupational Health and Safety (OHS) economic evaluation, one potential use is trade-off analysis between expected costs and benefits of an intervention when there is a budget constraint, or when one is interested in identifying the required budget to avert a specific number of lung cancer cases. For example, as shown in Table 1, we can consider a situation in which the budget is constrained to $70 million. In such a situation, using WM-LEV is the only intervention that will protect 100% of silica-exposed workers without the total intervention cost exceeding the pre-set amount. Trade-off analysis provides an opportunity for decision makers to define their targets regarding the prevention of a specific number of occupational lung cancer cases in the context of a predetermined budget.

To our knowledge, this is the first study to use the cost of silica-related occupational lung cancer cases averted for the benefit component in the economic evaluation of an intervention. Therefore, it is difficult to compare our findings with those of other studies. For example, Lahiri et al. [7] consider the averted cost of occupationally induced silicosis as a benefit and the cost of different interventions. They estimate the cost-effectiveness of interventions with a ratio (i.e., dollars per healthy years gained), and find they vary between $132.3 ($105.9 in 2005 US dollars) and $136.2 ($109 in 2005 US dollars) for different geographic sub-regions. However, they do not include cost items such as healthcare, informal caregiving, out-of-pocket, and home production losses in their analysis. Despite difference in economic evaluation methodologies and the inconsistencies of considered outcomes, our results are in line with Lahiri et al. [7], as we also identify the net benefit of WM-LEV as the highest among seven interventions. However, as they neither report the average per-case cost for interventions nor the number of silica-exposed workers affected, so we are unable to estimate a per-case value for their study.

In another study in the United States, OSHA estimates the net benefit of compliance with a new silica rule in terms of reduction of cost of silica-related diseases (i.e., fatal cases of lung cancer, non-malignant respiratory diseases, renal diseases and nonfatal cases of silicosis) [5]. They estimate the net annualized benefit of a reduction in the acceptable limit of exposure to be between $2.4 billion and $9.9 billion ($1.8 billion and $7.5 billion in 2009 US dollars), with a midpoint value of $6.1 billion ($4.6 billion in 2009 US dollars). Annually, the lowering of the exposure limit prevents 688 fatalities (567 fatalities in the construction sector) and 1585 moderate-to-severe silicosis cases (1080 cases in the construction sector).

In both of the studies referred to above, researchers assume all the variables are deterministic. However, uncertainty of variables is relevant for most of occupational health interventions. In an economic evaluation of an intervention one generally includes many variables with different levels of uncertainty, yet this uncertainty across data inputs has not been substantively addressed in OHS studies in the past. Ours is one of the first economic evaluation studies in OHS field to use BN. We also provide an overview of some of the potential benefits of using this approach and guidance on how to do so. Specifically, we explained the main steps of developing a BN in OHS setting, how to parametrize the variables, define the variable distribution, and incorporate them into a model. We capture the uncertainty of each variable and integrate the dependencies between them using a BN, and then estimate the expected net benefit of various interventions.

While the BN model developed in this study can support decision making, in its current form there is room for improvement of the approach. Future work in this area ought to include further research on the expansion of the model contents, including consideration of a broader set of variables. For example, in our study, the benefit side of our economic evaluation is limited to occupational lung cancer cases averted, despite the fact there are several other silica-related occupational diseases such as silicosis and silicosis-related diseases [4, 5, 7]. Additionally, to estimate averted productivity losses we focus only on absenteeism, not presenteeism, primarily because there is a lack of evidence to draw on for the magnitude of productivity losses associated with lung cancer cases upon return to work. Furthermore, our model structure can be improved upon by considering a greater number of relationships between the key variables, since we ignored some interactions because of limitations in background knowledge. For instance, interventions may adversely influence labour and/or equipment productivity [5], and under certain circumstances, the health-related quality of life of workers may be affected by the intervention [17]. Another example in this regard is the dependencies that might exist between age and sex in terms of the survival rates of occupational silica-related lung cancer cases. A more comprehensive analysis would consider other variable interactions that are caused by implementing an intervention. Moreover, in this study we do not investigate the time needed to implement silica reduction interventions and the duration of their effectiveness. Undoubtedly, because of the relatively long latency period of lung cancer, ultimate effect of silica reduction interventions will only be realized after several years. Further research is also needed on how to incorporate the uncertainty of the timeline of interventions into an economic evaluation, for example to account for variability in how many years after the introduction of an intervention it takes before the reduction of lung cancer cases reaches a steady state. Lastly, implementation of sensitivity analysis and sorting model variables by level of uncertainty is also one of the abilities wherein BN can provide invaluable analytic insight for policy makers, particularly for the purpose of developing data gathering strategies. In this regard, we also recommend implementation of Value of Information Analyses in future research, as it also enables one to identify parts of a model where additional data (reduction of uncertainty) is most useful.

## Conclusions

This study is one of the first to apply BN, as a probabilistic modeling approach, in the economic evaluation of an OHS intervention. It provides an overview of the potential advantages of the probabilistic modeling approach, in particular when decision contexts contain a large number of uncertain variables. Results indicate that, among seven silica exposure reduction interventions, the highest number of lung cancer cases are averted with a combined use of WM-LEV-PPE, but the highest net benefit is achieved with WM-LEV. Results also suggest that the level of exposure is an important determinant for the selection of the most cost-beneficial intervention. This study provides evidence that can assist researchers interested in demonstrating the monetary impact of decreasing or eliminating silica exposures in workplaces through various interventions. The positive return on investment of these interventions can help inform policy decision making, particularly in cases where optimal allocation of scarce resources is paramount. Considering the increasing attention being focused on the prevention of occupational cancer, we anticipate the case study provides important insights about silica exposure reduction interventions in the construction sector.

## Supplementary information

**Additional file 1.** Part A Bayesian Network Model for Economic Evaluation of Silica Exposure Reduction Interventions in Construction Sector. Part B Variables in the Economic Evaluation of Silica Exposure Reduction Interventions. Part C Silica-exposed Workers in the Construction Sector (Projection for 2020). Part D Secondary Silica Exposure. Part E Interventions Coverage. Part F Interventions Unit Cost. Part G Age Distribution of Lung Cancer Cases. Part H Probability of Lung Cancer in Different Level of Silica Exposure. Part I Direct, Indirect and Intangible Costs of Lung Cancer

## Data Availability

All data generated or analysed during this study are included in this published article [and its supplementary information files]. Cancer Care Ontario, Occupational Cancer Research Centre. Burden of occupational cancer in Ontario: Major workplace carcinogens and prevention of exposure. 2017. http://www.occupationalcancer.ca/wp-content/uploads/2017/09/Burden-of-Occupational-Cancer-in-Ontario.pdf. Accessed 26 Feb 2018. CAREX Canada. Silica (crystalline) occupational exposures. 2017. https://www.carexcanada.ca/en/silica_(crystalline)/occupational_estimate/#data_sources_and_methods. Accessed: Accessed 26 Feb 2018. Statistics Canada. Life Tables, Canada, provinces and territories 2010 to 2012. https://www150.statcan.gc.ca/n1/daily-quotidien/160519/dq160519c-eng.htm. Accessed 26 Feb 2018. Statistics Canada. Labour force survey, employment and unemployment, levels and rates, by province. 2017. http://www.statcan.gc.ca/tables-tableaux/sum-som/l01/cst01/labor07b-eng.htm. Accessed 26 Feb 2018. Statistics Canada: survey of labour and income dynamics. 2011. http://www.statcan.gc.ca/pub/75f0011x/75f0011x2013001-eng.htm. Accessed 26 Feb 2018. Statistics Canada. Sources of annual average growth in labour productivity in the total business sector. CANSIM Table 383–0021. 2017. http://www.statcan.gc.ca/pub/15-206-x/2013030/t001-eng.htm. Accessed 26 Feb 2018. Statistics Canada. General social survey cycle 24: time-stress and well-being public. 2017. http://gsg.uottawa.ca/data/teaching/eco/gssc24gid-ver4.pdf. Accessed 26 Feb 2018. Statistics Canada. Guide to the survey of employment, payrolls and hours. 2017. http://www.statcan.gc.ca/pub/72-203-g/72-203-g2017001-eng.htm. Accessed 26 Feb 2018. Statistics Canada. Canadian Community Health Survey. 2010. http://www23.statcan.gc.ca/imdb/p2SV.pl?Function=getSurvey&Id=81424. Accessed 26 Feb 2018.

## Abbreviations

BN: Bayesian network
CRMM: Canadian Cancer Risk Management Model
CSNA: Canadian System of National Accounts
GSS: General Social Survey
LEV: Local exhaust ventilation
LFS: Labour Force Survey
OCRC: Occupational Cancer Research Centre
OSHA: Occupational Safety and Health Administration
PPE: personal protective equipment
SLID: Survey of Labour and Income Dynamics
SPEH: Survey of Employment, Payrolls and Hours
CCHS: Canadian Community Health Survey
QALY: Quality-Adjusted Life Year
CCO: Cancer Care Ontario
WM: Wet method
