# Supplementary material for: A probabilistic approach for economic evaluation of occupational health and safety interventions: a case study of silica exposure reduction interventions in the construction sector
Source: BMC Public Health. 2020 Feb 11;20:210. doi: 10.1186/s12889-020-8307-7 (PMC7014628; doi:10.1186/s12889-020-8307-7)
Supplement: Supplementary file 1 — Additional file 1. Part A Bayesian Network Model for Economic Evaluation of Silica Exposure Reduction Interventions in Construction Sector. Part B Variables in the Economic Evaluation of Silica Exposure Reduction Interventions. Part C Silica-exposed Workers in the Construction Sector (Projection for 2020). Part D Secondary Silica Exposure. Part E Interventions Coverage. Part F Interventions Unit Cost. Part G Age Distribution of Lung Cancer Cases. Part H Probability of Lung Cancer in Different Level of Silica Exposure. Part I Direct, Indirect and Intangible Costs of Lung Cancer [file 12889_2020_8307_MOESM1_ESM.docx]

**ADDITIONAL FILE**

**A. Bayesian Network Model for Economic Evaluation of Silica Exposure Reduction Interventions in Construction Sector**


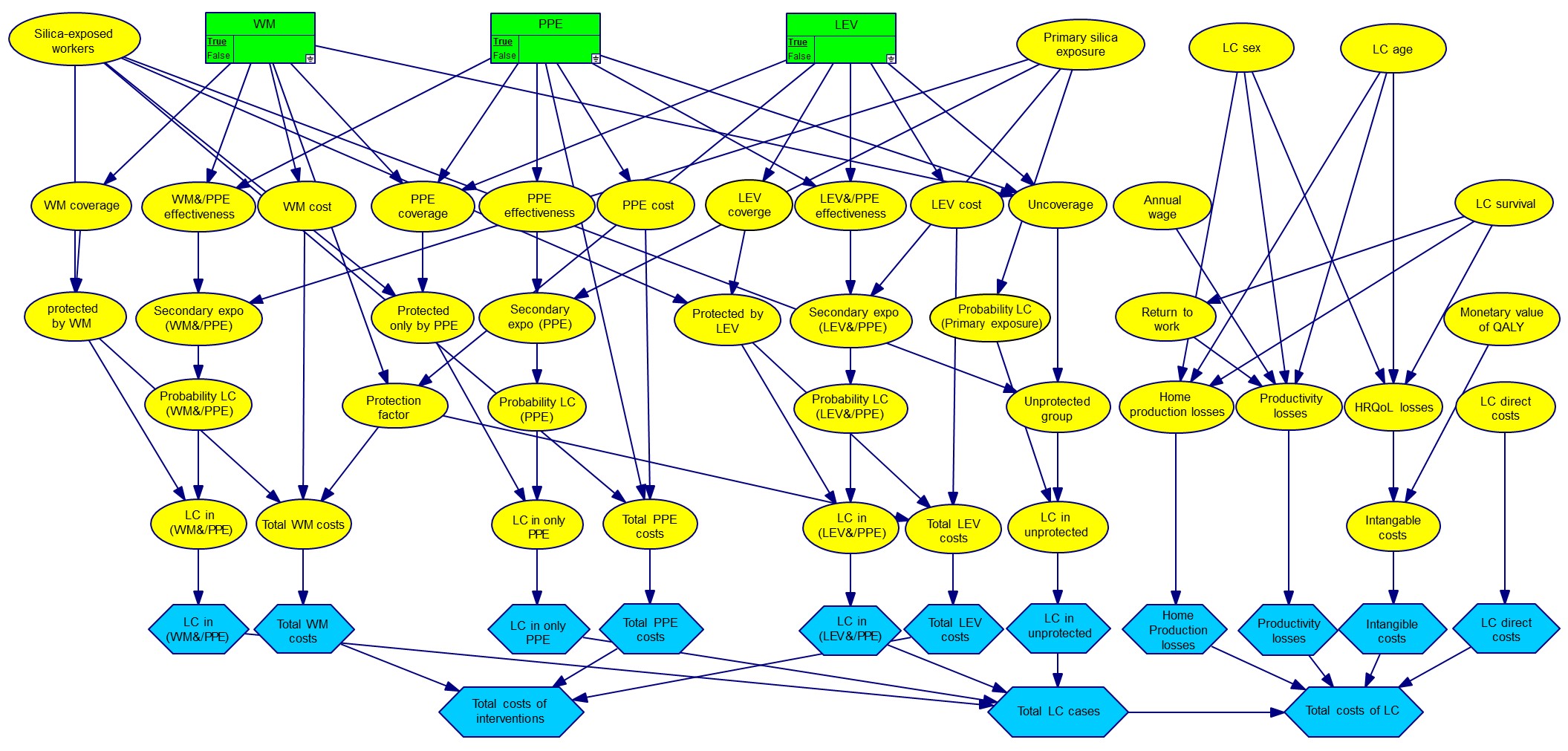
 ***Note****. Ellipses represent random variables, rectangles represent silica exposure reduction intervention options as decision variables, and hexagons represent costs as values or utility nodes, WM: wet method, LEV: local exhaust ventilation, PPE: personal protective equipment (decision node takes true if that intervention being implemented and take false if not), Coverage: percentage of silica-exposed workers that a specific intervention is applicable to them, Primary/secondary silica exposure: level of exposure to silica dust before/after an intervention, Protection factor: number of silica-exposed workers that can be protected by each unit of WM or LEV, LC in protected/unprotected groups: number of occupational lung cancer cases among protected/unprotected groups, LC survival: probability of survival of lung cancer, Return to work: rate of return to work of lung cancer cases after treatment, HRQoL losses: health-related quality of the life losses, LC direct costs: sum of healthcare, informal care, and out-of-pocket costs of lung cancer cases.*

**B. Variables in the Economic Evaluation of Silica Exposure Reduction Interventions**

| **Source** | **Range** | **State** | **Unit** | **Type** | **Variables** |
| --- | --- | --- | --- | --- | --- |
| OCRC^1^ | <64; 64-73; …; 118> | 7 | thousand | I^a^ | Silica-exposed workers |
| CAREX^2^ | low; medium; high | 3 | mg/m^3^ | L^c^ | Primary silica exposure^b^ |
| CRMM^3^ | survive; death | 2 |  | L | LC survival^d^ |
| OCRC^1^ | men; women | 2 |  | L | LC sex |
| OCRC^1^ | 25-29; 30-34; …; 85-99 | 13 | year | I | LC age |
| CRMM,^3^ Longo et al.^4^ Houtven et l.,^19^ | <59; 59-66; …; 101> | 7 | $ thousand | I | LC direct costs^e^ |
| LFS,^5^SLID,^6^ CSNA^7^ | 29; 36; 43 | 3 | $ thousand | N^q^ | Annual wage^f^ |
| Hirth et al.,^8^  Neumann et al.^9^ | 50; 80; …; 160; 200 | 6 | $ thousand | N | Monetary value of a QALY^g^ |
| OSHA^10^ | 0; 30;40;50; …; 80;100 | 7 | % | N | Intervention coverage^h^ |
| Lahiri et al.,^11^ Akbar-Khanzadeh et al.^12^ | 0; 0.6;0.7; …; 0.9;1 | 7 |  | N | Intervention effectiveness^i^ |
| This study | zero; low; medium; high | 4 | mg/m^3^ | L | Secondary silica exposure^j^ |
| This study | 0; 0-9; …;120-130 | 15 | thousand | I | (un)protected group |
| OCRC^1^ | 0;0-0.909; …;1.408-1.409 | 20 | 1E-3 | I | Probability of lung cancer^k^ |
| This study | 0; 0-10; …; 180-185 | 30 | case | I | LC in (un)protected^l^ |
| This study, OSHA^10^ | 0; 80; …; 23,980 | 5 | $ | N | WM unit cost^m^ |
| This study, OSHA^10^ | 0; 900; …; 6,000 | 5 | $ | N | LEV unit cost^n^ |
| This study, OSHA^10^ | 0-750; 750-800;…; 850> | 5 | $ | I | PPE unit cost^o^ |
| Lahiri et al.^11^ | 0; 1; …; 10 | 11 | person | N | Protection factor^p^ |
| This study | 0; 0-0.5; …; 2,000-3,000 | 20 | $ million | I | WM total costs |
| This study | 0; 0-0.8; …; 500-779 | 20 | $ million | I | LEV total costs |
| This study | 0; 0-30; …; 72-117 | 20 | $ million | I | PPE total costs |
| [Earle](https://www.ncbi.nlm.nih.gov/pubmed/?term=Earle%20CC%5BAuthor%5D&cauthor=true&cauthor_uid=20194860) et al.^13^ | 0; 77;100 | 3 | % | N | Return to work^r^ |
| This study | 0-2; 2-5; …; 1,439-1,445 | 26 | $ thousand | I | Productivity losses |
| GSS,^14^ SPEH^15^ | 0; 0-73; …; 741-790 | 20 | $ thousand | I | Home production losses |
| CCHS,^16^ CRMM^3^ | 0; 0-2.3; …; 16.4-16.9 | 10 | QALY^G^ | I | HRQoL losses^s^ |
| This study | 0-112; …; 3,860-4,070 | 20 | $ thousand | I | Intangible costs |

***Note.****^a^interval variable, ^b^level of exposure to silica dust before an intervention, ^c^labelled variable, ^d^survival probability of lung cancer, ^e^sum of healthcare, informal care, and out-of-pocket cost, ^f^adjusted annual wage, ^g^quality-adjusted life-years, ^h^percentage of silica-exposed workers are protected by each intervention, ^i^ability to reduce the level of exposure, ^j^level of exposure to silica dust after an intervention, ^k^probability of lung cancer among silica-exposed workers with different level of silica exposure, ^l^lung cancer cases among (un)protected group, ^m^implementation cost of wet method, ^n^impelemention cost of local exhaust ventilation, ^o^cost of personal protective equipment, ^p^number of silica-exposed workers that are protected by each unit WM or LEV, ^q^numbered variable, ^r^percentage of lung cancer cases return to work after treatment, ^s^health-related quality of the life losses. All monetary values are in 2017 Canadian dollars.*

**C. Silica-exposed Workers in the Construction Sector (Projection for 2020)**

| **Occupation description** | **NOC^a^** | **Eexposed^b^** |
| --- | --- | --- |
| Structural metal and platework fabricators and fitters | H323 | 19 |
| Civil engineering technologists and technicians | C131 | 9 |
| Artisans and crafts persons | F144 | 16 |
| Ironworkers | H324 | 23 |
| Automotive service technicians, truck mechanics and repairers | H421 | 35 |
| Glass forming and finishing machine operators and glass cutters | J123 | 47 |
| Machine operators, mineral and metal processing | J121 | 48 |
| Supervisors, mineral and metal processing | J011 | 50 |
| Janitors, caretakers and building superintendents | G933 | 103 |
| Crane operators | H621 | 167 |
| Light duty cleaners | G931 | 177 |
| Specialized cleaners | G932 | 260 |
| Drillers and blasters | H622 | 302 |
| Material handlers | H812 | 384 |
| Construction inspectors | C164 | 414 |
| Public works maintenance equipment operators | H612 | 487 |
| Other trades helpers and labourers | H822 | 527 |
| Concrete, clay and stone forming operators | J124 | 589 |
| Public works and maintenance labourers | H831 | 947 |
| Insulators | H143 | 2,530 |
| Tile setters | H133 | 2,840 |
| Truck drivers | H711 | 2,858 |
| Contractors-supervisors, heavy construction equipment crews | H017 | 3,254 |
| Concrete finishers | H132 | 4,198 |
| Contractors and supervisors, other construction trades, installers, repairers and servicers | H019 | 5,156 |
| Heavy equipment operators (except crane) | H611 | 6,592 |
| Plumbers | H111 | 7,062 |
| Bricklayers | H131 | 10,071 |
| Plasterers, drywall installers, finishers, and lathers | H134 | 10,153 |
| Construction trades helpers and labourers | H821 | 31,772 |
| **Sum** |  | 91,089 |

***Note.*** *^a^national occupational code in Canada, ^b^number of silica-exposed workers in Ontario, Canada construction sector estimated for 2020 based on Occupational Cancer Research Centre (OCRC) methodology. For more details regarding this methodology readers refer to the main reference.^24^ The probability distribution for variable silica-exposed workers assumed to follow Gaussian distribution SD=μ/10.^17^ (i.e. in seven intervals of less than 64, 64-73, 73-82, 82-91, 91-100, 100-109, 109-118 thousend workers).*

**D. Secondary Silica Exposure**

Expression 1 estimate the level of silica exposure after implementation of intervention by considering primary silica exposure and the effectiveness of each intervention. C_1_ is the primary silica exposure (i.e. level of exposure before impelemention of interventions), E_x_ is the intervention effectiveness . x is the silica exposure reduction intervention, which can takes WM, LEV, PPE, or combination of them. Note that for combined use of engineering controls (WM and LEV) with PPE, we consider an additive effects, which means the escaped silica dust from WM or LEV, is captured by PPE. C_2_ is secondary silica exposure (i.e. level of exposure after impelemention of interventions).

| C_2_ = $\left\{ \begin{aligned} \text{(1-}\text{E}_{\text{x}}\text{) × }\text{C}_{\text{1}}\text{ Intervention is }\text{impelemented } \\ \text{C}_{\text{1}}\text{ Intervention is not-implemented } \end{aligned} \right.$ | (1) |
| --- | --- |

**D1. Intervention Effectiveness**

| **Intervention** (Y=component implemented; N=component not implemented) | **WM=Yes** | | **WM=No** | | **LEV=Yes** | | **LEV=No** | |
| --- | --- | --- | --- | --- | --- | --- | --- | --- |
|  | **PPE =Y** | **PPE= N** | **PPE =Y** | **PPE= N** | **PPE =Y** | **PPE= N** | **PPE =Y** | **PPE= N** |
| **Effectivenes rang**^a^ |  |  |  |  |  |  |  |  |
| 0 | 0.00 | 0.040 | 0.021 | 1 | 0.003 | 0.017 | 0.021 | 1 |
| 0.6 | 0.01 | 0.135 | 0.077 | 0 | 0.010 | 0.064 | 0.077 | 0 |
| 0.7 | 0.04 | 0.277 | 0.192 | 0 | 0.034 | 0.167 | 0.192 | 0 |
| 0.8 | 0.10 | 0.304 | 0.284 | 0 | 0.085 | 0.268 | 0.284 | 0 |
| 0.85 | 0.18 | 0.178 | 0.249 | 0 | 0.155 | 0.260 | 0.249 | 0 |
| 0.9 | 0.22 | 0.056 | 0.129 | 0 | 0.210 | 0.154 | 0.129 | 0 |
| 1 | 0.44 | 0.010 | 0.048 | 0 | 0.502 | 0.070 | 0.048 | 0 |

***Note.*** *^a^for WM-PPE, LEV-PPE, we consider the additive effects, which means the escaped silica from WM or LEV, is captured by PPE. But we assume the combined use of WM and LEV does not change the overall effectiveness, as they protect different occupations in the construction sector. effectiveness of interventions assumed to follow Gaussian distribution with SD=μ/10.^17^* *based on the central limit theorem. This theory assumes regardless of the population distribution model, as the sample size increases, the sample mean tends to be normally distributed around the population mean, and its standard deviation shrinks as n increases.*

**E. Interventions Coverage**

Expression 2 estimates the number of silica-exposed workers that are protected by each intervention, or protected group. x is the silica exposure reduction intervention, which can takes WM, LEV, PPE, or combination of them. We drew the WM and LEV coverage from OSHA^10^, as they identified which of these interventions are applicable for which occupational codes in construction sector. To used their data, we first match silica-exposed national occupations codes (NOC) in the Canadian construction sector with occupational employment statistics (OES) code in the United States that OSHA has identified. Then we estimate number of silica-exposed workers that are protected by each intervention. We assume silica-exposed workers that are protected by each intervention is not changed in case of combined use of WM and LEV, as these interventions protect different occupational codes. In case of using WM-PPE, certain number of silica-exposed workers are protected by both intervention (i.e. 60%) and the rest are only protected by PPE (i.e. 40%) (similarly are defined for LEV-PPE intervention). PPE coverage is estimated silica-exposed workers that are exclusively protected by PPE. In another word, in case of using PPE with any other intervention, only the portion of the workers that exclusively are covered with PPE, are considered here. Table E1 represented the conditional probability table of this variable.

| (Un)Protected group_(x)_= number of silica-exposed workers × intervention coverage_(x)_ | (2) |
| --- | --- |

**E1. PPE Coverage Conditional Probability Table**

| **Intervention** (Y=component implemented; N=component not implemented) | **WM= Y** | | | | **WM=N** | | | |
| --- | --- | --- | --- | --- | --- | --- | --- | --- |
|  | **LEV= Y** | | **LEV=N** | | **LEV= Y** | | **LEV=N** | |
|  | **PPE=Y** | **PPE=N** | **PPE=Y** | **PPE=N** | **PPE=Y** | **PPE=N** | **PPE=Y** | **PPE=N** |
| **Coverage (%)** |  |  |  |  |  |  |  |  |
| 0 | 1 | 1 | 1 | 0 | 1 | 0 | 1 | 0 |
| 30 | 0 | 0 | 0 | 0 | 0 | 0 | 0 | 0 |
| 40 | 0 | 0 | 0 | 1 | 0 | 0 | 0 | 0 |
| 50 | 0 | 0 | 0 | 0 | 0 | 0 | 0 | 0 |
| 60 | 0 | 0 | 0 | 0 | 0 | 1 | 0 | 0 |
| 80 | 0 | 0 | 0 | 0 | 0 | 0 | 0 | 0 |
| 100 | 0 | 0 | 0 | 0 | 0 | 0 | 0 | 1 |

**F. Interventions Unit Cost**

**F1. Wet Method (WM)**

| **Unit cost**^a^ | **Probability** | **WM is applicable** |
| --- | --- | --- |
| $80 | 0.13 | Masonry and concrete cutters using portable saws - I |
| $710 | 0.38 | Millers using portable or mobile machines |
| $3,100 | 0.37 | Jackhammers and powered chipping tools, Rock crushing machine operators and tenders, Underground (tunnel) construction work |
| $23,980 | 0.12 | Heavy construction equipment operators (demolition, abrading, fracturing), Heavy construction equipment operators (earthmoving) |

***Note.****^a^including three sub-categories of purchase of equipment, maintenance, and operating cost. Values are in 2017 Canadian dollars.*

**F2. Local Exhaust Ventilation (LEV)**

| **Unit cost**^a^ | **Probability** | **LEV is applicable** |
| --- | --- | --- |
| $900 | 0.04 | Masonry and concrete cutters using portable saws |
| $1,150 | 0.77 | Tuck pointers and grinders (hand-held) |
| $1,400 | 0.09 | Hole drillers using held-held or stand-mounted drills |
| $6,000 | 0.1 | Rock and concrete drilling |

***Note.****^a^including three sub-categories of purchase of equipment, maintenance, and operating cost. Values are in 2017 Canadian dollars.*

**F3. Personal Protective Equipment (PPE)**

| **Unit cost**^a^ | **Probability** | **PPE is applicable** |
| --- | --- | --- |
| $0-$750 | 0.20 | All occupations in construction sector |
| $750-$800 | 0.21 |  |
| $800-$850 | 0.43 |  |
| > $850 | 0.16 |  |

***Note.*** *^a^assuming average yearly per worker cost of PPE at $820, based on more frequently used silica half-mask cost, which includes costs of respirator and filters at $714, shaving kit at $118, fit testing at $33, cleaning at $60, and training at $13. Values are in 2017 Canadian dollars. PPE unit cost assumed to follow Gaussian distribution with SD=μ/10.^17^*

**G. Age Distribution of Lung Cancer Cases**^1^

**H. Probability of Lung Cancer in Different Level of Silica Exposure**

| **Level of silica-exposure** | **Number of Silica-exposed workers**^a^ | **Silica-related lung cancer cases**^b^ | **Probability of silica-related lung cancer**^c^ | **Alpha**^d^ | **Beta**^d^ |
| --- | --- | --- | --- | --- | --- |
| Low | 47,179 | 43 | 9.1E-04 | 1.2E+08 | 1.3E+11 |
| Medium | 38,701 | 45 | 1.2E-03 | 7.5E+07 | 6.4E+10 |
| High | 13,825 | 19 | 1.4E-03 | 5.0E+07 | 3.6E+10 |

***Note.****^a^number of silica-exposed workers in Ontario, Canada construction sector based on Occupational Cancer Research Centre (OCRC) projection.^1^ ^b^estimated incidence of silica-induced lung cancers in different level of silica exposure (They used a modelling approach to approximates the proportion of total cancer that are attributed to silica exposure. For more information regarding the estimation methodology readers refer to the main reference), ^c^average probability of lung cancer among silica-exposed workers by dividing the number of the silica-related lung cancer cases by number of silica-exposed workers. ^d^probability of silica-related lung cancer assumed to follow beta distribution with α=μ^2^/s^2^ and β=s^2^/μ.^17^*

**I. Direct, Indirect and Intangible Costs of Lung Cancer**^18^

**I1. Direct costs**

| **Direct cost**^a^ | **Probability** | **Direct cost**^a^ | **Probability** |
| --- | --- | --- | --- |
| <$59 | 0.001 | $84-$93 | 0.341 |
| $59-$65 | 0.021 | $93-$101 | 0.136 |
| $65-$76 | 0.136 | >$101 | 0.023 |
| $76-$84 | 0.341 |  |  |

***Note.****^a^direct cost of lung cancer includes three subcategories of healthcare, informal caregiving and out-of-pocket cost. We identify the statistical average healthcare costs for lung cancer cases over the entire treatment paradigm at $42 thousand based on CRMM.^3^ Out-of-pocket costs are estimated at $24 thousand based on and Longo et al.^4^ and informal caregiving costs are estimated $17 thousand.^19^ For estimation of informal caregiving costs, we assume 16 hours of caregiving per week at the weighted average provincial minimum wage.^15^ Values are in 2017 Canadian thousand dollars.*

**I2. Indirect Costs**

**I2.1 Output/Productivity Losses**

Expression 3 estimate the output/productivity losses of construction workers as a result of poor health or premature mortality, similar to the study undertaken with Tompa et al.^18^ This expression depends on the age of lung cancer cases at diagnosis in comparison to general population, their survival rate, annual wage, and return to work rate. We define return to work rate following treatment of lung cancer cases at 0.77 similar to Earle et al.^13^ We also assume once cases return to work, their productivity is same as the general population productivity. For lung cancer survivors, we compare the survival probabilities of the general Canadian population with lung cancer cases and for fatalities cases, we estimate the wage of the individual and the amount of work time is lost due to premature death. In this expression α is the number of years during which health will be compromised for cancer (here 10 years maximum), and µ represents the upper limit of years over which the computation is to be estimated. We include a productivity growth of 1% in projections to estimate the value of the future labour-market activity. Monetary values beyond the reference year are discounted to 2017 Canadian dollars, using a discount rate of 3% per year.

| Output/Productivity losses of lung cancer cases=  $\left\{ \begin{aligned} \sum_{\text{n=0}}^{\text{α}} \frac{\text{(}\text{survival probability of lung cancer cases}\text{ }\text{× (1-return to work rate)×}{\text{ }\text{annual wage}\text{)}}_{n}}{{\text{(1+ }\text{discount rate}\text{)}}^{\text{n}}}\text{ }\text{Survivors}\text{ } \\ \sum_{\text{n=0}}^{\mu} \frac{\text{ (}{survival probability of the general population \times annual wage\text{)}}_{n}}{\text{(}{\text{1}\text{+ }\text{discount rate}\text{)}}^{\text{n}}} \mathrm{Fatalities} \end{aligned} \right.$ | (3) |
| --- | --- |

**I2.2 Home Production Losses**

Expression 4 is used to estimate the potential home production losses.^18^ we extracted the average time that Canadians spend on various household-related activities (e.g. taking care of plants and animals, food cooking, home clean-up, maintenance, and other personal activities) from the General Social Survey (GSS)^14^ and monetized it using the minimum wage of housekeepers for each age and sex group using Survey of Employment, Payrolls and Hours (SPEH).^15^ Our model only includes home production losses of premature mortality cases.

| Home production losses of lung cancer cases=  $\sum_{\text{n=0}}^{\mu} \frac{\text{ (}{survival probability of general population \times\mathrm{household}time \times housekeepers wage\text{)}}_{n}}{\text{(}\text{1+ discount rate)}^{\text{n}}}$ | (4) |
| --- | --- |

**I3. Intangible Cost**

Expression 5 estimate the losses of health-related quality of the life due to poor health or premature mortality,^18^ by comparing the health-related quality of the life of lung cancer cases^3^ (Table I3.1) with the health-related quality of the life of general population^16^ (Table I3.2). α represent number of years during which health will be compromised for cancer cases, and µ is the upper limit of years over which the computation is to be estimated.

| Health-related quality of life losses of lung cancer cases=  $\left\{ \begin{aligned} \sum_{\text{n=0}}^{\text{α}} \frac{{{(survival \mathrm{probability} of population\times HRQoL}_{G}-{\mathrm{survival}\mathrm{probability} of lung cancer\times HRQoL}_{P})}_{n}}{{\text{(1+}\text{discount rate}\text{)}}^{\text{n}}}\text{ Survivors } \\ \sum_{\text{n=0}}^{\mu} \frac{{{(survival \mathrm{probability} \mathrm{general} population\times HRQoL}_{G})}_{n}}{\text{(}{\text{1+}\text{discount rate}\text{)}}^{\text{n}}}\mathrm{Fatalities} \end{aligned} \right.$ | (5) |
| --- | --- |

**I3.1 Health Utility Index Losses for Lung Cancer Cases**

| **Year after diagnosis** | **1** | **2** | **3** | **4** | **5** | **6** | **7** | **8** | **9** | **10** |
| --- | --- | --- | --- | --- | --- | --- | --- | --- | --- | --- |
| Reduced health utility index^a^ | 0.640 | 0.704 | 0.731 | 0.742 | 0.751 | 0.755 | 0.760 | 0.762 | 0.764 | 0.778 |

***Note.*** *^a^ten-year weighted average survival probabilities for lung cancer cases (weighted by stage/survival for both sexes), from cancer risk management model (CRMM).^3^*

**I3.2 Health Utility Index for General Population**^a^

| **Age** | **20-24** | **25-29** | **30-34** | **35-39** | **40-44** | **45-49** | **50-54** | **55-59** | **60-64** | **65-69** | **70-74** | **75-79** | **80-84** |
| --- | --- | --- | --- | --- | --- | --- | --- | --- | --- | --- | --- | --- | --- |
| Men | 0.92 | 0.92 | 0.92 | 0.92 | 0.90 | 0.89 | 0.88 | 0.86 | 0.88 | 0.85 | 0.84 | 0.82 | 0.71 |
| Women | 0.92 | 0.92 | 0.92 | 0.91 | 0.90 | 0.87 | 0.87 | 0.85 | 0.85 | 0.85 | 0.82 | 0.78 | 0.72 |

***Note****. ^a^Canadian community health survey (CCHS), 2010.^16^*

**REFERENCES**

1 Cancer Care Ontario, Occupational Cancer Research Centre. Burden of occupational cancer in Ontario: Major workplace carcinogens and prevention of exposure. 2017. Available at: http://www.occupationalcancer.ca/wp-content/uploads/2017/09/Burden-of-Occupational-Cancer-in-Ontario.pdf. Accessed: February 26, 2018.

2 CAREX Canada. Silica (Crystalline). 2017. Available at: https://www.carexcanada.ca/en/silica_(crystalline)/occupational_estimate/#data_sources_and_method. Accessed: February 26, 2018.

3 Evans WK, Wolfson MC, Flanagan WM, et al. Canadian cancer risk management model: evaluation of cancer control. Int J Technol Assess Health Care. 2013;29:131-9.

4 Longo CJ, Fitch M, Deber RB, et al. Financial and family burden associated with cancer treatment in Ontario, Canada. Support Care Cancer. 2006;14:1077-85.

5 Statistics Canada. Labour force survey, employment and unemployment, levels and rates, by province. Available at: http://www.statcan.gc.ca/tables-tableaux/sum-som/l01/cst01/labor07b-eng.htm. Accessed: February 26, 2018.

6 Statistics Canada: survey of labour and income dynamics. Available at: http://www.statcan.gc.ca/pub/75f0011x/75f0011x2013001-eng.htm. Accessed: February 26, 2018.

7 Statistics Canada. Sources of annual average growth in labour productivity in the total business sector. CANSIM table 383-0021. Available at: http://www.statcan.gc.ca/pub/15-206-x/2013030/t001-eng.htm. Accessed: February 26, 2018.

8 Hirth RA, Chernew ME, Miller E, et al. Willingness to pay for a quality-adjusted life year: in search of a standard. Med Decis Making. 2000;20:332-42.

9 Neumann PJ, Cohen JT, Weinstein MC. Updating cost-effectiveness—the curious resilience of the $50,000-per-QALY threshold. N Engl J Med Overseas Ed. 2014;371:796-7.

10 US Occupational Safety and Health Administration. Final economic analysis and final regulatory flexibility analysis. supporting document for the final rule for occupational exposure to respirable crystalline silica. 2016. Available at: https://www.federalregister.gov/documents/2016/07/26/2016-17270/occupational-exposure-to-respirable-crystalline-silica-approval-of-collections-of-information. Accessed: February 26, 2018.

11 Lahiri S, Levenstein C, Nelson DI, et al. The cost effectiveness of occupational health interventions: prevention of silicosis. Am J Ind Med. 2005;48:503-14.

12 Akbar-Khanzadeh F, Milz SA, Wagner CD, et al. Effectiveness of dust control methods for crystalline silica and respirable suspended particulate matter exposure during manual concrete surface grinding. J Occup Environ Hyg. 2010;7:12.

13 Earle CC, Chretien Y, Morris C, et al. Employment among survivors of lung cancer and colorectal cancer. J Clin Oncol. 2010;28:1700.

14 Statistics Canada. General social survey cycle 24: time-stress and well-being public. Available at: http://gsg.uottawa.ca/data/teaching/eco/gssc24gid-ver4.pdf. Accessed: February 26, 2018.

15 Statistics Canada. Guide to the survey of employment, payrolls and hours. Available at: http://www.statcan.gc.ca/pub/72-203-g/72-203-g2017001-eng.htm. Accessed: February 26, 2018.

16 Statistics Canada. Canadian Community Health Survey. 2010. Available at: http://www23.statcan.gc.ca/imdb/p2SV.pl?Function=getSurvey&Id=81424. Accessed: February 26, 2018.

17 Briggs A, Sculpher M, Claxton K. Decision modelling for health economic evaluation. New York, Oxford University Press, 2006:77-120.

18 Tompa E, Kalcevich C, McLeod C, et al. The economic burden of lung cancer and mesothelioma due to occupational and para-occupational asbestos exposure. J Occup Environ Med. 2017;74:816-22.

19 Van Houtven CH, Ramsey SD, Hornbrook MC, et al. Economic burden for informal caregivers of lung and colorectal cancer patients. Oncologist. 2010;15:883-93.
